# Transcobalamin II deficiency mimicking myelodysplastic syndrome in a child: a case report

**DOI:** 10.3389/fped.2026.1757303

**Published:** 2026-04-10

**Authors:** Xiangrong Hu, Huiping Xu, Linjun Xie

**Affiliations:** 1 The First Hospital of Putian City, Putian, China; 2 The School of Clinical Medicine, Fujian Medical University, Fuzhou, China

**Keywords:** bicytopenia, inheritedmetabolic disorders, pediatric hematology, TCN2, transcobalamin II deficiency

## Abstract

Transcobalamin II (*TCN2*) deficiency is a rare autosomal recessive metabolic disorder that impairs vitamin B12 transport and can present with megaloblastic anemia and neutropenia, often mimicking hematologic diseases such as myelodysplastic syndrome (MDS). We report a 5-year-old male presenting with pallor, fatigue, and fever. On admission, he had acute anemia crisis and neutropenia, with chest CT revealing a lung infection, while serum folate and vitamin B12 levels were normal. Bone marrow smears and flow cytometry suggested possible MDS; however, genetic testing identified compound heterozygous pathogenic variants in the *TCN2* gene, confirming *TCN2* deficiency. Following parenteral methylcobalamin therapy, his symptoms improved and blood counts gradually normalized. Oral maintenance therapy was then initiated, with stable hematologic parameters during follow-up. This case highlights the importance of considering rare metabolic disorders such as *TCN2* deficiency in pediatric patients with unexplained anemia and neutropenia, and underscores the value of early recognition, genetic diagnosis, and long-term management. These findings contribute to the limited literature on pediatric *TCN2* deficiency and reinforce clinician awareness when evaluating children with MDS-like presentations.

## 1 Introduction

Transcobalamin II (*TCN2*) deficiency is an extremely rare congenital disorder affecting cobalamin metabolism, first reported in 1971 (1). It is an autosomal recessive condition caused by pathogenic variants in the *TCN2* gene. Despite normal or even elevated serum vitamin B12 levels, the absence of functional *TCN2* impairs cellular uptake of cobalamin, resulting in intracellular deficiency (2). *TCN2* is a critical plasma transport protein that delivers cobalamin to tissues via receptor-mediated endocytosis, playing a key role in cellular metabolism and DNA synthesis (3). The true prevalence may be underestimated, with current estimates suggesting fewer than 1 in 1,000,000 live births are affected (4). Early recognition and intervention are crucial to prevent severe anemia, immunodeficiency, and neurological complications (5).

Patients with *TCN2* deficiency typically present during infancy with a range of clinical manifestations. The most common features include failure to thrive, pancytopenia, vomiting, anemia, and diarrhea (6). Hematologic abnormalities arise from impaired DNA synthesis due to disrupted methylcobalamin and adenosylcobalamin pathways. Clinically, the disorder may mimic severe combined immunodeficiency, leukemia, myelodysplastic syndrome (MDS), or inherited bone marrow failure syndromes, making early and accurate diagnosis challenging (2, 4, 7).

Here, we report a pediatric patient with *TCN2* deficiency who initially presented with MDS-like hematologic features, highlighting the importance of considering rare metabolic disorders in the differential diagnosis of pediatric cytopenias. Unlike most reported cases, successful long-term maintenance was achieved with oral methylcobalamin, which may provide a potential approach for long-term management. Informed consent was obtained from the patient's parents.

## 2 Case description

### 2.1 Clinical presentation

A 5-year-and-10-month-old boy was admitted with a one-week history of pallor, fatigue, and fever. He had a history of recurrent respiratory infections and had previously been seen multiple times in outpatient and inpatient settings at our hospital. Eighteen months before our first observation, the patient was diagnosed with *α*-thalassemia trait through genetic testing. Both he and his father were found to be heterozygous for the Southeast Asian (–SEA) deletion of the *α*-globin gene cluster (*α*α/−−SEA). There was no other known family history of hematologic or metabolic disorders.

### 2.2 Physical examination

At admission, the patient weighed 14.5 kg (−2 SD) and measured 108 cm in height (−2 SD). On examination, the axillary temperature was 39.8 °C, the heart rate 128 beats per minute, the blood pressure 92/62 mm Hg, and the respiratory rate 27 breaths per minute. The patient showed signs of malnutrition, including emaciation, pale complexion, pallid lips, thin subcutaneous fat, and poor skin turgor. Angular stomatitis was also observed. No lymphadenopathy was noted. Lung examination revealed coarse breath sounds with bilateral moist rales, and the liver and spleen were not palpable. Psychomotor development and neurological examination were unremarkable.

### 2.3 Laboratory examinations

Complete blood count revealed severe anemia and leukopenia: red blood cell count (RBC) 1.98 × 10^12^/L (4.3–5.8), hemoglobin (Hb) 43 g/L (130–175), mean corpuscular volume (MCV) 71.2 fL (82–100), red blood cell distribution width (RDW) 86.5 fL (37–54), white blood cell count (WBC) 1.96 × 10^9^/L (3.5–9.5), absolute neutrophil count (ANC) 0.48 × 10^9^/L (1.6–7.8), absolute lymphocyte count (ALC) 1.31 × 10^9^/L (1.5–4.6), and platelet count 182 × 10^9^/L (125–350). Peripheral blood smear showed anisocytosis predominantly with macrocytes, along with target cells, stomatocytes. Biochemical tests revealed total protein 52.6 g/L (60–85), globulin 14.7 g/L (20–38), and lactate dehydrogenase (LDH) 735 U/L (80–258). Other biochemical parameters, including liver and renal function tests, electrolytes, and blood glucose, were within normal limits. Serum ferritin was 185.3 ng/mL (30.06–400.2), folate 13.74 ng/mL (4.2–19.02), vitamin B12 539 pg/mL (180–900), C-reactive protein 35.94 mg/L (0–8), and procalcitonin 0.117 ng/mL (0–0.05). Urinalysis was unremarkable, fecal occult blood test was negative, and the direct Coombs test was negative. Chest CT indicated bronchiectasis with superimposed infection. Detailed laboratory findings are summarized in Table 1.

**Table 1:** Laboratory findings at presentation.

Parameter	Reference Range, Children, This Hospital^a^	At Presentation
Red blood cell count (RBC) (×10^12^/L)	4.3–5.8	1.98
Hemoglobin (Hb) (g/L)	130–175	43
Mean corpuscular volume (MCV) (fL)	82–100	71.2
Red cell distribution width (RDW) (fL)	37–54	86.5
White blood cell count (WBC) (×10^9^/L)	3.5–9.5	1.96
Absolute neutrophil count (ANC) (×10^9^/L)	1.6–7.8	0.48
Absolute lymphocyte count (ALC) (×10^9^/L)	1.5–4.6	1.31
Platelet count (×10^9^/L)	125–350	182
Total protein (g/L)	60–85	52.6
Globulin (g/L)	20–38	14.7
Lactate dehydrogenase (LDH) (U/L)	80–258	735
Serum ferritin (ng/mL)	30.06–400.2	185.3
Folate (ng/mL)	4.2–19.02	13.74
Vitamin B12 (pg/mL)	180–900	539
C-reactive protein (mg/L)	0–8	35.94
Procalcitonin (ng/mL)	0–0.05	0.117
Direct Coombs test	-	Negative

^a^ Reference ranges are based on age-matched pediatric populations in our institution and may vary depending on patient characteristics and laboratory methods; therefore, they may not be applicable to all patients.

### 2.4 Bone marrow examination

**Figure 1:**
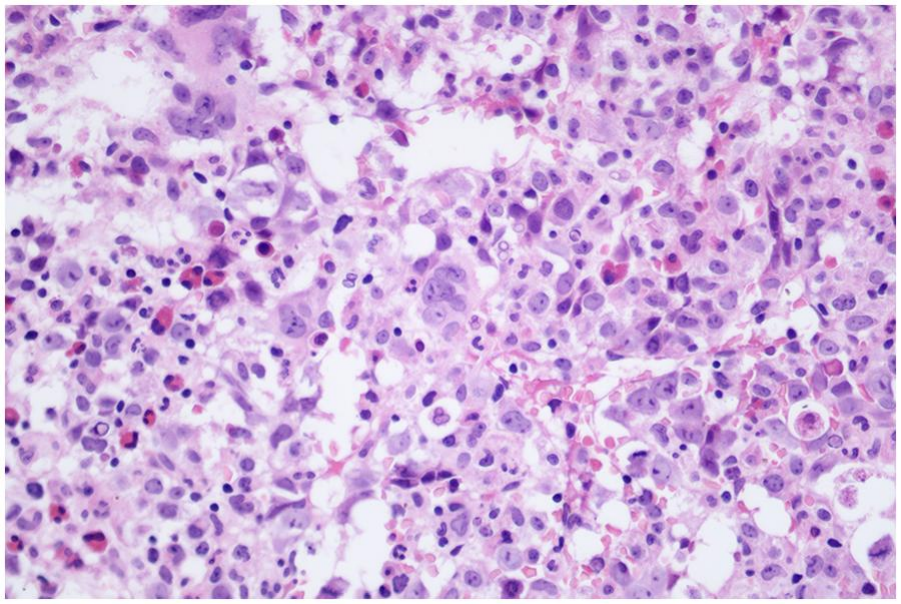
Bone marrow biopsy of the patient. Bone marrow histology shows overall normal cellularity with increased erythroid lineage and some megaloblastic changes (H&E staining,   ×  400).

Bone marrow smear showed markedly active nucleated cell proliferation. Myeloid blasts accounted for 3.5%, with prominent megaloblastic changes in the granulocytic series. Erythroid lineage showed slightly decreased proliferation, predominantly intermediate-to-late erythroblasts, with megaloblastic changes, Howell-Jolly bodies, nuclear budding, pyknosis, and binuclear or multinuclear forms, suggesting multilineage dysplasia. Flow cytometry revealed 2.72% myeloid blasts with decreased or absent HLA-DR expression and abnormal phenotypes; reduced granulocyte proportion with abnormal CD13/CD16 expression; erythroid cells with increased FSC and SSC, and 2.6% immature monocytes, not excluding MDS. Cytogenetic analysis was normal, and a 34-gene panel for myeloid malignancies was negative. Bone marrow histopathology demonstrated overall normal cellularity with increased erythroid proportion and some megaloblastic changes, warranting differentiation between megaloblastic anemia and MDS (see Figure 1).

### 2.5 Genetic analysis

**Figure 2:**
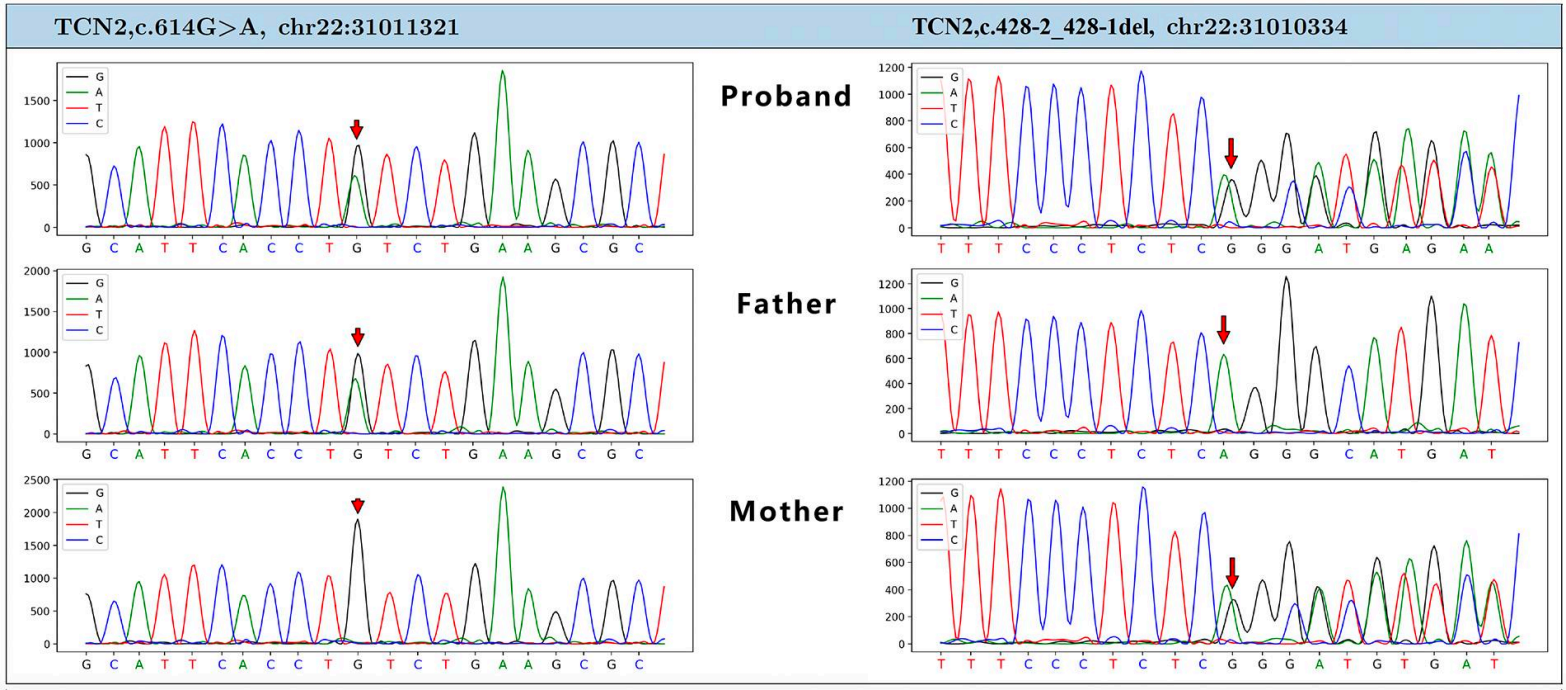
Genetic analysis of the patient. Sanger sequencing revealed compound heterozygous variants in the *TCN2* gene: a maternally inherited c.428-2_428-1del and a paternally inherited c.614G > A (*p*.Cys205Tyr).

To support the clinical diagnosis, we performed whole-exome sequencing (WES) followed by Sanger sequencing validation. The results revealed that the patient carried compound heterozygous variants in the *TCN2* gene (NM_000355.3): a c.428-2_428-1del variant inherited from the mother and a c.614G > A (p.Cys205Tyr) variant inherited from the father (see Figure 2). The c.428-2_428-1del variant is located in the intronic region of the *TCN2* gene and is a splice site mutation that disrupts normal mRNA splicing, leading to defects in protein synthesis. On the other hand, the c.614G > A variant is located in exon 5 of the *TCN2* gene, causing a missense mutation, where the 205th amino acid in the protein is replaced from cysteine (Cys) to tyrosine (Tyr), thereby disrupting the structure and function of the protein. These genetic findings confirm the diagnosis of *TCN2* deficiency.

### 2.6 Treatment

Initially, the patient received transfusions of packed red blood cells and antibiotics, which led to defervescence. Bone marrow smear and flow cytometry raised suspicion of MDS; however, high-frequency myeloid mutation screening was negative, and the karyotype was normal. Considering the possibility of an inherited metabolic disorder, whole-exome sequencing was performed, leading to a definitive diagnosis of *TCN2* deficiency. The patient was treated daily with intramuscular injections of methylcobalamin (1 mg), resulting in significant improvement of blood counts. After discharge, methylcobalamin was continued at 1 mg once weekly via intramuscular injection. Three months later, Hb had risen to 104 g/L, WBC to 5.92 × 10^9^/L, ANC to 2.15 × 10^9^/L, and ALC to 3.09 × 10^9^/L. At the request of the patient's family, therapy was switched to oral methylcobalamin (0.5 mg/day) for maintenance.

### 2.7 Retrospective review and follow-up

**Figure 3:**
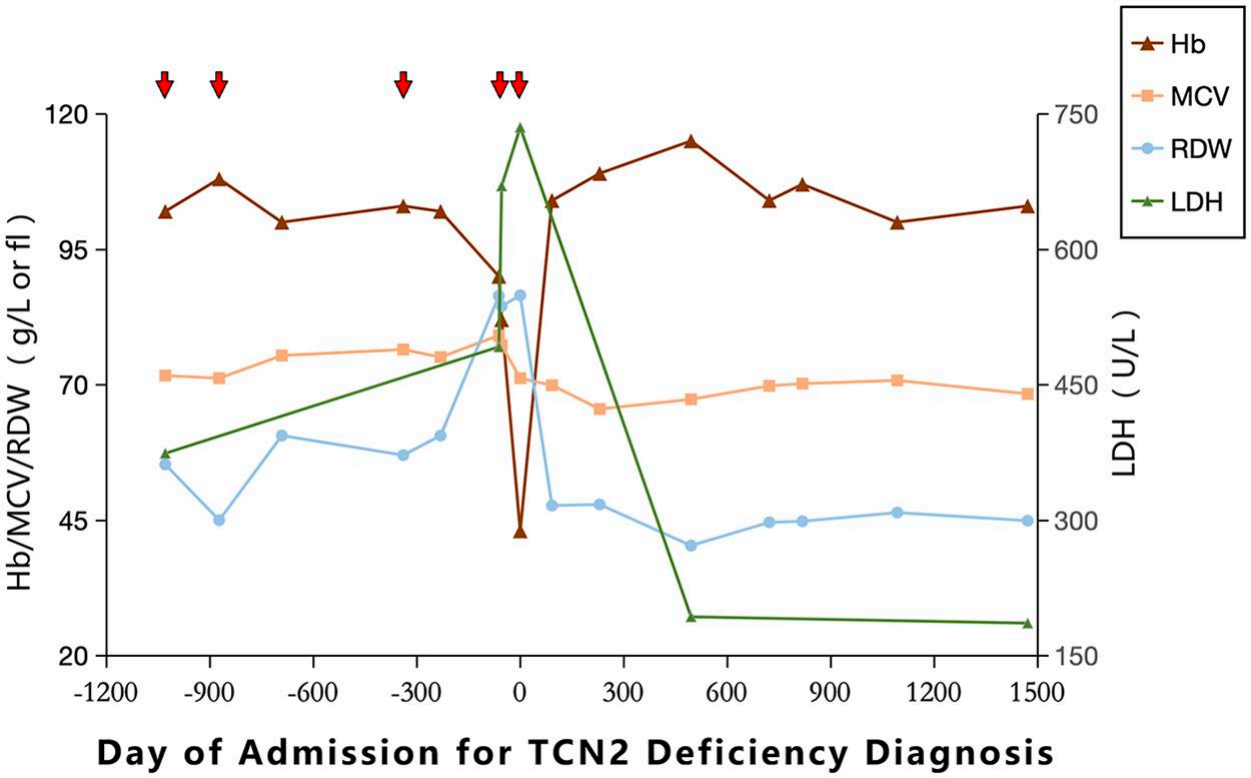
Changes in blood parameters over time, with the red arrows indicating each hospitalization event.

A retrospective review of the patient's medical history revealed a long-standing mild anemia, with genetic testing at the time identifying *α*-thalassemia trait due to heterozygosity for the Southeast Asian (–SEA) deletion of the *α*-globin gene cluster (*α*α/−−SEA). However, his average mean corpuscular volume (MCV, 75.2 fL) and red cell distribution width (RDW, 64.2 fL), along with markedly elevated lactate dehydrogenase (LDH, mean 512 U/L), were inconsistent with isolated *α*-thalassemia trait. Following treatment, MCV and RDW decreased and LDH returned to normal; these parameters remained stable after switching to oral methylcobalamin for maintenance therapy (see Figure 3). Prior to treatment, the patient had been hospitalized four times for recurrent respiratory infections; afterward, the family reported a marked reduction in infections, with no further hospital admissions for respiratory illness. His height also improved from −2 SD below the mean for Chinese children to the normal range (at the time of writing, age 9 years 10 months, height 136 cm).

## 3 Discussion

Bicytopenias in children arise from a broad differential that includes infections, nutritional deficiencies, immune-mediated destruction, drug or toxin exposures, marrow infiltration, inborn errors of immunity (IEI), and inherited metabolic as well as bone marrow failure syndromes (8). In this case, the acute onset of severe anemia and neutropenia, coupled with dysplastic features on bone marrow examination, initially raised concern for MDS. Pediatric MDS is rare, affecting 1–4 per million children annually, with nearly half of cases associated with congenital or germline predispositions. Therefore, before establishing a diagnosis of *de novo* MDS, a comprehensive evaluation is required to exclude alternative causes, including metabolic disorders, IEI, and inherited bone marrow failure syndromes.

In our patient, the bone marrow findings were not consistent with inherited bone marrow failure syndromes. Given the history of recurrent infections and decreased globulin levels, metabolic disorders and IEI were included in the differential diagnosis. WES was subsequently performed, which identified *TCN2* deficiency, and no pathogenic variants associated with IEI were detected. Considering the limitations of this technique, IEIs cannot be completely excluded; however, based on the clinical features and the favorable response to treatment, the likelihood of an underlying IEI was considered to be very low. Pongphitcha et al. reported a child with a homozygous *TCN2* c.428-2A > G mutation who presented with pancytopenia, hepatosplenomegaly, recurrent infections, acute hemolytic crises, and circulating immature myeloid cells (7). Similarly, Rana et al. described a case of *TCN2* deficiency with severe cytopenias, increased marrow blasts, and both morphological and chromosomal abnormalities (9). Like these reports, our case mimicked MDS, highlighting the importance of considering rare metabolic disorders in the differential diagnosis of pediatric patients with MDS-like hematologic abnormalities.

A retrospective review of our patient's early clinical course revealed a missed opportunity for earlier recognition. The patient's previous measurements showed relatively elevated MCV, markedly increased RDW, and persistently elevated LDH. These findings deviate significantly from the typical presentation of isolated *α*-thalassemia trait, which is characterized by microcytosis, stable RDW, and normal or only mildly elevated LDH when hemolysis is absent. Instead, these discordant parameters strongly suggest a combined megaloblastic anemia process. Such discordant parameters should prompt early evaluation. Because serum vitamin B12 levels are typically normal in *TCN2* deficiency, assessment of methylmalonic acid and homocysteine levels or genetic testing may facilitate timely diagnosis (2). In addition, following treatment, the patient experienced fewer infections, and his height returned to the population average. These findings further support that early intervention may enhance immune function and promote normal growth (5, 6).

*TCN2* deficiency, caused by homozygous or compound heterozygous pathogenic variants, typically presents in early infancy (10, 11). According to Engin Kose et al., among the 53 reported cases of Transcobalamin II deficiency, the age at diagnosis ranged from birth to 12 months, with most cases identified within the first 2–3 months of life (6). Thus, compound heterozygosity does not necessarily indicate a milder phenotype or later onset, but rather reflects individual variability in symptom expression. Guryanova et al. reported a 5-year-old initially admitted for an acute anemia crisis who was ultimately diagnosed with *TCN2* deficiency; after treatment, hemoglobin normalized, although mild cognitive impairment persisted (12). In our patient, growth and development had been mildly delayed since birth, nutritional status was suboptimal, and he experienced angular stomatitis and recurrent respiratory infections, eventually leading to bronchiectasis. These findings highlight the importance of recognizing non-hematologic features of *TCN2* deficiency.

The *TCN2* variants identified in our patient were identical to those reported by Lim et al. (13), namely the pathogenic splicing variant c.428-2_428-1delAG and the variant of uncertain significance c.614G > A; p.(Cys205Tyr). Lim et al. demonstrated through an *in vitro* mRNA splicing assay that c.428-2_428-1delAG leads to an in-frame deletion of 153 nucleotides in exon 4 (*TCN2*:r.428_580del), resulting in the predicted loss of 51 amino acids (*TCN2*:p.Gly143_Val193del) and abrogating two of the three cobalamin-binding sites. This experiment confirmed that the variant completely abolishes normal full-length protein production from the affected allele. Meanwhile, c.614G > A; p.(Cys205Tyr) affects a highly conserved residue essential for one of the intramolecular disulfide bonds of transcobalamin and is predicted to impair protein stability and function. These mechanistic insights from Lim et al. provide a plausible molecular basis for the pathogenicity of the same combination of variants observed in our patient.

Parenteral hydroxocobalamin or methylcobalamin remains the first-line therapy for *TCN2* deficiency (6). Prompt and sufficient parenteral B12 supplementation can rapidly reverse cytopenias, diarrhea, and immune dysfunction, supporting normal growth and development (5, 6, 14). However, in some cases, persistent neurologic or growth deficits may occur, often due to delayed diagnosis or inadequate therapy, highlighting the importance of early intervention (15–18). In this patient, parenteral methylcobalamin led to swift recovery of blood counts, confirming that timely treatment effectively restores hematopoietic function.

After hematologic parameters stabilized, long-term maintenance therapy remained necessary, as discontinuation could lead to disease recurrence (2, 4, 15, 18). Although intramuscular injection is the commonly used route, it may reduce adherence in pediatric patients. Oral vitamin B12 supplementation offers a convenient alternative for maintenance therapy but requires careful monitoring, including regular assessment of blood counts, metabolic markers (such as methylmalonic acid and homocysteine), immune function, and growth (19). This study evaluated the effectiveness of oral maintenance therapy in this patient. The results showed that after switching to oral methylcobalamin, the patient's blood counts remained within the normal range, providing a novel option for long-term management.

## 4 Conclusion

This case highlights the diagnostic challenges of identifying *TCN2* deficiency in pediatric patients presenting with MDS-like hematologic abnormalities. Careful evaluation of laboratory trends may reveal underlying metabolic defects, genetic testing is essential for establishing a definitive diagnosis. Timely recognition and parenteral vitamin B12 supplementation can rapidly restore hematologic function, prevent complications, and enable stable long-term management, including oral maintenance therapy under close monitoring. Overall, this case emphasizes the importance of timely diagnosis, genetic confirmation, and appropriate long-term management strategies.

## Data Availability

The original contributions presented in the study are included in the article/Supplementary Material, further inquiries can be directed to the corresponding author.
